# Supplementary material for: Accurate and Rapid Genetic Tracing the Authenticity of Floral Originated Honey with the Molecular Lateral Flow Strip
Source: Biosensors (Basel). 2022 Nov 4;12(11):971. doi: 10.3390/bios12110971 (PMC9688305; doi:10.3390/bios12110971)
Supplement: Supplementary file 1 [file biosensors-12-00971-s001.zip › biosensors-1949261-supplementary.pdf]

Supporting Information

Accurate and Rapid Genetic Tracing the Authenticity of Floral Originated Honey with the Molecular Lateral Flow Strip

Qian Wu[1,†], Qi Chen[1,†], Chao Yan[1, 2], Jianguo Xu[1], Zhaoran Chen[2], Li Yao[1], Jianfeng Lu[1], Bangben Yao[2,*] and Wei Chen[1, 3, *]

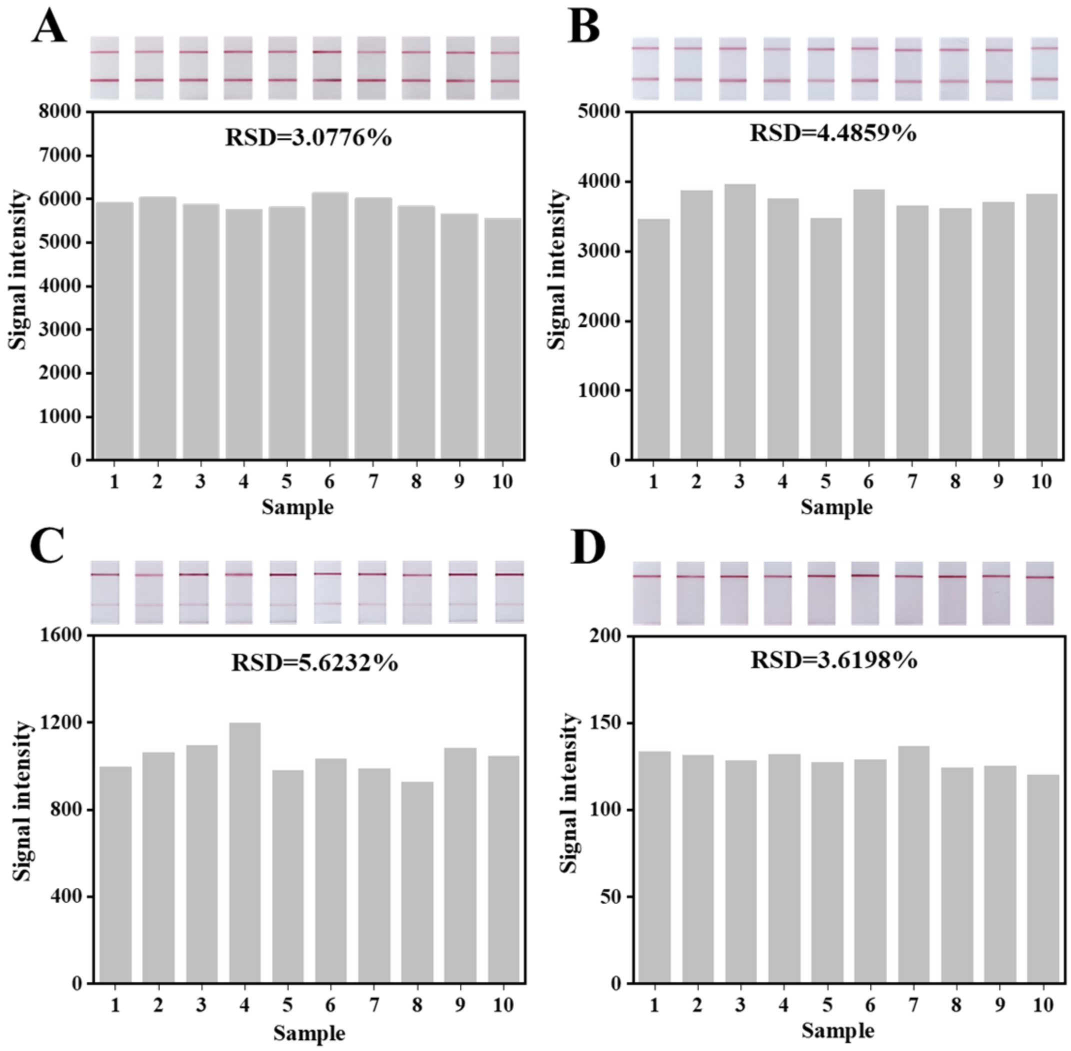

Figure S1. The detection results of the 10 honey samples adulterated at different ratio of (A) 100%; (B) 75%; (C) 25% and (D) 0% (v/v%) with the PLFS.

Table S1. The results of real honey sample analysis by the qPCR method (Cq value > 40 means adulteration) and PLFS method.

Sample	1	2	3	4	5	6	7
qPCR Cq value	29.08	26.79	27.93	24.97	26.39	24.57	28.55
PLFS Qualitative	Partial adulteration	Partial adulteration	Partial adulteration	Without adulteration	Partial adulteration	Without adulteration	Partial adulteration
Sample	8	9	10	13	12	13	14
qPCR Cq value	28.86	25.45	21.90	>40	25.53	>40	>40
PLFS Qualitative	Partial adulteration	Partial adulteration	Without adulteration	Adulteration	Partial adulteration	Adulteration	Adulteration
